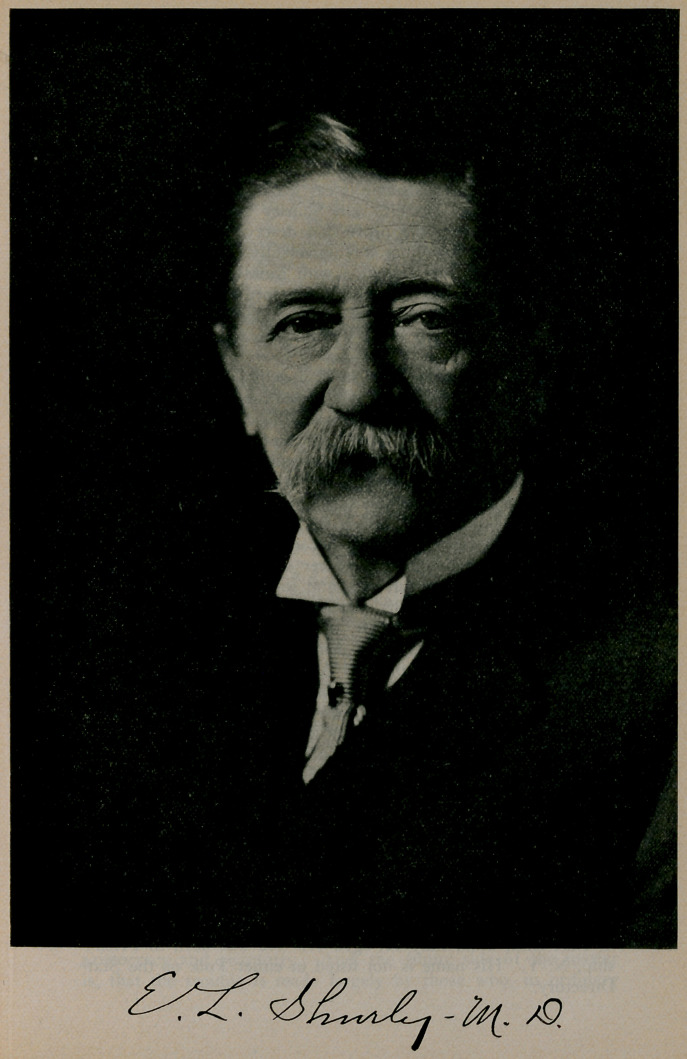# Dr. E. L. Shurley

**Published:** 1913-06

**Authors:** 


### OBITUARIES.

*Readers are requested to report promptly the death of any physician in Western New York, or former residents of this region, or graduates of any medical school in Western New York and to call the attention of the families of the deceased, our desire to publish adequate obituary notices.*

Dr. E. L. Shurley, Buffalo, 1866, died suddenly of heart disease at the Harper Hospital of Detroit, May 17, aged 67. He was born in Buffalo, but went to Detroit in 1872, having had an experience as army surgeon in the Indian campaigns of the late 60's, where he distinguished himself for his bravery. His military service terminated with the Yellowstone compaign of 1870, and he then practiced in Manistee for a couple of years. In Detroit he soon made a name for himself as a laryngologist, his contributions to the therapy of tuberculosis being especially noteworthy. It is a curious coincidence that Dr. Keeley heard

E. L. Shurly - M. D.

his lecture in Chicago on the treatment of tuberculosis with the chlorid of gold and sodium, just before announcing his cure for alcoholism. Dr. Shurley was well known as an author, and held the presidency of the American Laryngological Association and the Michigan State Medical Society, besides being active in various other organizations. Dr. Shurley was a widower and left no children, but his name is carried on by his nephew, Dr. Burt R. Shurley of Detroit. Dr. Shurley not only enjoyed the wide acquaintance in the profession deserved by his contributions to medical science, but was universally esteemed and loved. He always kept up his affiliation with Buffalo, having contributed to this journal, delivered lectures before the Buffalo profession and maintained a personal acquaintance with many of the Buffalo profession. His death will be long felt.

## Figures and Tables

**Figure f1:**